# Psychological Stress Accompanied by a Low-Variety Diet Is Positively Associated with Type 2 Diabetes in Middle-Aged Adults

**DOI:** 10.3390/nu12092612

**Published:** 2020-08-27

**Authors:** Yoonjin Shin, Yangha Kim

**Affiliations:** Department of Nutritional Science and Food Management, Ewha Womans University, Seoul 03760, Korea; yjin19@hotmail.com

**Keywords:** psychological stress, type 2 diabetes, dietary variety score, low-variety diet

## Abstract

Psychological stress is generally known to affect dietary intakes and cause chronic diseases. This study aims to investigate the association between psychological stress and the risk of type 2 diabetes in relation to dietary variety. A total of 126,405 middle-aged adults were enrolled in the Korean Genome and Epidemiology Study. Stress levels were measured using the psychosocial well-being index. Dietary variety score (DVS) was defined as the number of different foods consumed over a day. Stress levels were positively associated with the risks of diabetes (odds ratio (OR) for tertile 3 compared with tertile 1, men: OR = 1.34 (95% CI: 1.24–1.45); women: OR = 1.29 (1.19–1.4)). As the stress levels rose, the intake of grains increased, and the intake of fruits and vegetables decreased. Participants with higher stress levels showed lower DVS than those with lower stress levels. Furthermore, participants with higher stress levels and lower DVS had a significantly higher OR for diabetes than those with lower stress levels and higher DVS (men: OR = 1.83 (1.58–2.12); women: OR = 1.85 (1.59–2.51)). These results suggest that the high risk of type 2 diabetes for people with high stress levels may be associated with low dietary variety.

## 1. Introduction

Psychological stress is widely used to indicate the negative emotional, behavioral, and biological response to a perceived threat. A small amount of stress can be desirable, beneficial, and even healthy [1]. However, excessive or prolonged psychological stress may influence the pathogenesis of physical disease. Stress induces chronic activation of the neuroendocrine pathways that secrete adrenaline, noradrenaline, and cortisol. These hormones affect the body’s sensitivity to insulin secretion and may interfere with normal blood sugar control, leading to diabetes [2].

Type 2 diabetes is a growing global health problem, and the number of people with diabetes is expected to double between 2000 and 2030 [3]. In Korea, the prevalence of diabetes in individuals aged 30 years has increased from 8.6% in 2001 to 11.0% in 2013 [4]. Due to prolonged lifespan, the incidence of diabetes is expected to increase more than previously anticipated. The first-line therapy for individuals with diabetes is known as lifestyle modification, involving diet [5]. Therefore, to reduce diabetes epidemics, individual efforts are needed to determine the modifiable factors that can affect their lifestyle and eating habits.

Dietary variety refers to the total number of different food items that are consumed individually for a certain period of time and is a major aspect of diet quality [6]. Eating a more varied diet is known to be related to higher nutritional adequacy; thus, several dietary guidelines have highlighted the value of eating a variety of foods [7,8]. According to a previous study, a greater dietary variety is related to a lower intake of refined grains and a higher intake of fruits and vegetables [9]. Moreover, a greater dietary variety was reported to have a protective effect against chronic diseases, including metabolic syndrome, cardiovascular disease, and cancer [10,11,12]. Therefore, dietary variety is supposed to improve nutritional status and reduce the risk of disease.

According to a 35-year follow-up study of middle-aged Swedish men, perceived stress is associated with a higher incidence of type 2 diabetes [13]. In addition, a Japanese cohort of men and women showed that the risk of diabetes increased by 50% in relation to perceived stress during a 10-year follow-up [14]. Collectively, these studies support the positive association between chronic psychological stress and the risk of diabetes. However, to our knowledge, no previous study has evaluated whether stress status may affect dietary variety and its relation to the prevalence of diabetes. Therefore, we aim to evaluate the association between stress and risk of diabetes in relation to dietary variety among middle-aged adults who participated in the Korean Genome and Epidemiology Study (KoGES).

## 2. Materials and Methods

### 2.1. Study Population

KoGES is a consortium project of population-based cohort studies aimed at investigating the genetic and environmental risk factors of common complex diseases found in the Korean population [15]. KoGES includes the Health Examinees (HEXA) Study and the Cardiovascular Disease Association Study (CAVAS), which consist of participants aged 40 years at baseline who were recruited from the national health examinee registry. We combined the data of the HEXA study (*n* = 173,357) and CAVAS (*n* = 28,338), conducted from 1 January 2004 to 31 December 2013. Initially, 201,695 individuals were recruited at baseline. Among them, only middle-aged adults (*n* = 174,131) were included in the study. Participants who did not complete the assessment of stress levels (*n* = 13,498), who did not have biochemical profiles (*n* = 5183), or who reported implausibly low or high intakes of energy (<500 or 4000 kcal/day; *n* = 3074) were excluded. Participants who had self-reported diagnoses of cancer, cardiovascular disease, liver disease, and stomach disease were also excluded (*n* = 6628). Thus, 126,279 middle-aged adults (42,945 men and 83,334 women) were included in our final analyses. This study was approved by the Institutional Review Board of Ewha Womans University (No. 2019-0003, January 2019), and the procedures were performed in accordance with the Helsinki Declaration of 1975, as revised in 2008.

### 2.2. Assessment of Stress Levels

The psychological stress level of participants was measured using the psychological well-being index short form (PWI-SF) based on the General Health Questionnaires [16] and developed by Chang [17]. Its reliability and validity have been established for Korean laborers and the general public [17]. Respondents score each item using a four-point Likert scale, ranging from “strongly disagree” (0) to “strongly agree” (3); their scores were summed to determine the levels of stress, with higher scores indicating higher stress levels. In this study, participants were divided into three groups based on the tertile points of the score.

### 2.3. Assessment of Dietary Intake

Well-trained interviewers obtained the dietary data using a semiquantitative food-frequency questionnaire (SQFFQ) at the survey sites, including national and international medical schools, hospitals, and health institutions. Each participant provided the frequency of their average intake of 106 types of foods over a 12-month period (on a 9-point scale, ranging from “never” to ≥3 servings/d) and the average intake quantity (on a 3-point scale: “less” (0.5 serving), a standard amount (1 serving), or “more” (1.5 servings)) according to questions from SQFFQ, developed and validated by KoGES [18,19]. Daily average food and nutrient intakes were calculated using a weighted frequency per day and a portion size per unit. The nutrient database of the Korean Nutrition Society was used to convert food intake into nutrients [20].

### 2.4. Dietary Variety Score

To evaluate the diversity of food intake, the dietary variety score (DVS) was calculated by counting the number of different food items consumed over a day by a nutritionist. The method was originally devised by Randall et al. [21], and this study modified the method reported by Choi et al. [22]. Food consumed several times during the period was counted only once. Each time another food is added, the DVS is increased by 1 point. Foods with the same main ingredients, such as pork belly and pork roast, were considered one food. The possible totals (*n* = 86) reflect all kinds of foods currently consumed in this study.

### 2.5. Classification of Diabetes

Type 2 diabetes was defined as a self-reported previous diagnosis by a physician, use of oral hypoglycemic agents or insulin, or a fasting glucose concentration of ≥126 mg/dl based on the World Health Organization criteria [23].

### 2.6. General Characteristics, Anthropometric Measurements, and Biochemical Variables

Data on demographic characteristics, current drinker (yes, no), current smoker (yes, no), moderate physical activity (yes, no), education (<high school, ≥high school), monthly income (<2 million KRW, ≥2 million KRW), and history of disease were obtained using an interview-administered questionnaire. Height was measured with a stadiometer, and weight was measured using a metric system while the participants were wearing a patient gown with no shoes. Body mass index (BMI) was calculated as weight as kilograms divided by height in meters squared. Waist circumference (cm) was measured half-way between the lowest rib margin and the iliac crest using a nonstretchable standard tape. Blood pressure was measured at least twice on the right arm using an automatic sphygmomanometer or standardized mercury. Blood samples were collected after fasting for more than 8 h, and plasma concentrations of high sensitive C-reactive protein (hs-CRP), total cholesterol, HDL cholesterol, triacylglycerol (TG), and glucose were quantified using the enzyme method (ADVIA 1650 and ADVIA 1800; Siemens Healthineers, Deerfield, IL, USA). For participants with TG concentrations <400 mg/dL, LDL cholesterol was calculated as described by Friedewald [24].

### 2.7. Statistical Analysis

All statistical analyses were performed using SAS v9.4 (SAS Institute Inc., Cary, NC, USA), and *p*-values of 0.05. Continuous variables were expressed as mean ± standard error and categorical variables as percentages. The participants were categorized into tertiles for relative comparison of stress levels according to the PWI score. The general linear model and the Cochran–Mantel–Haenszel analysis were used to determine the differences in means and distribution of general characteristics and to test the linear trends according to stress levels. Variables that were statistically significant in univariate analysis or known as potentially important factors associated with dietary intake and stress-induced chronic disease were considered potential confounders and adjusted in the analysis. Multivariate logistic regression analysis was applied to obtain ORs and 95% CIs for the risk of diabetes. Dietary intakes were adjusted for total energy intake using the residual method.

## 3. Results

The participants’ general characteristics according to stress levels are shown in Table 1. Men with higher stress levels tended to be slightly younger and had lower BMI than those with lower stress levels. On the contrary, women with higher stress levels were older and had a higher BMI. Men and women with higher stress levels tended to be earning less income, less educated, less likely to exercise, and more likely to drink and smoke. In men and women, those with higher stress levels exhibited higher concentrations of hs-CRP, fasting glucose, and HBA1c than those with lower stress levels.

Higher stress levels were associated with an increased prevalence of diabetes (Table 2). In multivariate analyses, men with the highest stress levels had higher odds of diabetes than those with the lowest stress levels (OR for tertile 3 compared with tertile 1, OR = 1.34 (95% CI: 1.24–1.45) *p*-trend < 0.0001). Women with the highest stress levels also showed higher odds of diabetes (OR = 1.29 (95% CI: 1.19–1.40) *p*-trend < 0.0001).

Men and women with higher stress levels showed higher carbohydrate intakes than those with lower stress levels, but all other nutrient intakes were lower (Table 3). As stress levels increased, consumption of grains increased while consumption of fruits, vegetables, and protein foods decreased. Furthermore, men and women with higher stress levels showed a lower DVS than those with lower stress levels.

The prevalence and ORs of diabetes according to stress levels and DVS are presented in Table 4. In multivariate analyses, men and women with higher stress levels and a lower DVS had significantly higher odds of diabetes than those with lower stress levels and a higher DVS (men: OR = 1.83 (95% CI: 1.58–2.12); women: OR = 1.85 (95% CI: 1.59–2.51)).

## 4. Discussion

This study investigates the association between psychological stress and the risk of type 2 diabetes in relation to dietary variety. The risk of diabetes was higher in participants with higher stress levels than those with lower stress levels. Participants with higher stress levels showed a higher intake of grains but a lower intake of fruits and vegetables than those with lower stress levels. Stress levels were associated with decreased DVS. Moreover, participants with higher stress levels and lower DVS had significantly higher ORs for diabetes than those with lower stress levels and higher DVS.

Stress has been associated with the development of type 2 diabetes [13,25,26]. Chronic activation of the hypothalamic–pituitary–adrenal axis due to long-term stress may interfere with cortisol release regulation. Cortisol stimulation has a direct impact on insulin sensitivity and reduces insulin secretion. Job strain, a form of psychological stress in the workplace, has been related to the high risk of type 2 diabetes in adults of European cohort studies [27]. Our study also shows that stress is associated with the risk of diabetes. To reduce the risk of diabetes, it is necessary to examine the possible modifiable factors, such as dietary habits, that are known to affect it.

As consuming a variety of food is known to be positively associated with the maintenance of health, dietary guidelines have long emphasized the value of eating a variety of foods [7,8]. Moving from a monotonous to a diverse diet can increase the probability of nutrient adequacy [28]. In this study, dietary variety was lower in participants with higher stress levels than those with lower stress levels. Stress is known to induce the activation of the sympathetic adrenal medulla system, along with the release of adrenaline and noradrenaline, which suppress appetite [29]. As appetite decreases, it can be assumed that the amount and number of food consumed decreases. Moreover, the reduction in the number of foods consumed is associated with a decrease in dietary diversity. This result is consistent with a prior study in which stress was inversely associated with dietary variety, which indicates the quality of a meal [30].

Lower dietary variety has been associated with higher intakes of refined grains and lower intakes of fruits and vegetables [9]. We found that participants with higher stress levels consumed more grains and had fewer fruits and vegetables. This finding indicates that stressful situations can lead to less consumption of side dishes and simple meals based on staple foods such as rice, bread, and noodles, thus increasing the intake of grains while reducing the intake of other foods. This diet increases carbohydrate intake while reducing the intake of all other nutrients, including fiber and antioxidant vitamins.

Excessive intake of carbohydrates and low fiber and antioxidants may aggravate glucose intolerance and increase the risk of diabetes. Rapidly absorbed carbohydrates, characterized by a high glycemic load, may aggravate glucose intolerance and increase metabolic disorder [31]. Consumption of high carbohydrate diets has been associated with high levels of fasting glucose and the risk of diabetes mellitus in Korean women [32]. In contrast, antioxidants nutrients are known to have a protective effect against the risk of diabetes by inhibiting the peroxidation chain reaction [33]. According to a prospective cohort study, vitamin C intake was significantly lower in cases of type 2 diabetes [34]. Fiber can also control or lower serum glucose by delaying glucose entry into the bloodstream and reducing the post-meal rise of serum glucose [35].

Stress has been suggested to be a risk factor for the development of diabetes. Stress is also often thought to be a consequence of diabetes. Diabetes patients have reported higher stress and greater depressive symptoms than healthy controls [36]. According to a meta-analysis, people with diabetes are two times more likely to have depression than people without diabetes [37]. It is known that the diagnosis of diabetes or the burden of dealing with its complications may lead to psychological stress [38]. This evidence suggests that the association between stress and diabetes is potentially bidirectional. However, because our analysis was cross-sectional, we could not determine the temporality of this association, and we are limited to discussing the aspects of diabetes risk due to stress.

The present study has several limitations. First, whether stress is related to DVS and the risk of diabetes was analyzed using a cross-sectional study, and the cause–effect could not be established. Second, we used SQFFQ to estimate the participant’s dietary intake, which could be an inaccurate method of quantifying the exact amounts of food consumed. Well-trained interviewers with a validated SQFFQ and pictures of portion sizes conducted the survey, but measurement error in dietary intake seems inevitable [19]. Nevertheless, to our knowledge, this is the first study to examine the association between stress and the risk of type 2 diabetes in relation to dietary variety.

## 5. Conclusions

The present study used data from KoGES to demonstrate that high levels of psychological stress are associated with the risk of type 2 diabetes in Korean adults. Highly stressed individuals with a low variety of dietary intake showed a higher OR for diabetes compared with those with lower stress levels and higher DVS. Therefore, psychological stress accompanied by a low-variety diet is partially related to diabetes risk.

## Figures and Tables

**Table 1:** General characteristics of the subjects by tertiles of stress levels.

Variables	Men				Women			
	T1 (Lowest)	T2 (Intermediate)	T3 (Highest)		T1 (Lowest)	T2 (Intermediate)	T3 (Highest)	
	(*n* = 15,562)	(*n* =12,637)	(*n* = 14,746)	*p*-Trend	(*n* = 28,227)	(*n* = 28,132)	(*n* = 26,975)	*p*-Trend
Stress levels (score)	7.9 ± 0.03	13.7 ± 0.01	23.0 ± 0.04	<0.0001	8.9 ± 0.02	15.7 ± 0.01	26.1 ± 0.03	<0.0001
Age (years)	52.0 ± 0.06	51.4 ± 0.06	51.3 ± 0.06	<0.0001	51.0 ± 0.04	50.9 ± 0.04	51.2 ± 0.04	0.020
Height (cm)	168.9 ± 0.05	169.0 ± 0.05	168.7 ± 0.05	<0.0001	156.7 ± 0.03	156.6 ± 0.03	156.2 ± 0.03	<0.0001
Weight (kg)	70.4 ± 0.07	70.1 ± 0.08	69.5 ± 0.08	<0.0001	58.1 ± 0.04	58.1 ± 0.05	58.1 ± 0.05	0.927
Waist circumstance (cm)	86.1 ± 0.06	85.7 ± 0.07	85.6 ± 0.06	<0.0001	78.4 ± 0.05	78.4 ± 0.05	78.9 ± 0.05	<0.0001
Body mass index (kg/m^2^)	24.6 ± 0.02	24.5 ± 0.02	24.4 ± 0.02	<0.0001	23.6 ± 0.02	23.7 ± 0.02	23.8 ± 0.02	<0.0001
Systolic blood pressure (mmHg)	126.0 ± 0.12	125.9 ± 0.13	125.7 ± 0.12	0.241	120.8 ± 0.09	120.4 ± 0.09	120.7 ± 0.10	0.276
Diastolic blood pressure (mmHg)	79.3 ± 0.08	79.2 ± 0.09	79.8 ± 0.08	<0.0001	75.0 ± 0.06	74.9 ± 0.06	75.3 ± 0.06	0.0004
hs-CRP (mg/dL)	0.31 ± 0.01	0.30 ± 0.01	0.42 ± 0.01	<0.0001	0.21 ± 0.01	0.21 ± 0.01	0.28 ± 0.01	<0.0001
HBA1c (%)	5.7 ± 0.01	5.7 ± 0.01	5.8 ± 0.01	<0.0001	5.6 ± 0.01	5.7 ± 0.01	5.7 ± 0.01	<0.0001
Glucose (mg/dL)	98.9 ± 0.19	99.2 ± 0.21	100.2 ± 0.22	<0.0001	92.5 ± 0.11	92.8 ± 0.11	93.4 ± 0.13	<0.0001
Current drinker (%)	11,600 (74.6)	9700 (76.8)	11,133 (75.6)	0.001	9351 (33.2)	9241 (32.9)	9475 (35.2)	<0.0001
Current smoker (%)	4871 (31.3)	4388 (34.8)	5895 (40.0)	<0.0001	472 (1.7)	604 (2.2)	934 (3.5)	<0.0001
Moderate exercise (%)	9167 (59.0)	6857 (54.3)	6786 (46.1)	<0.0001	15,384 (54.6)	13,909 (49.5)	11,019 (40.9)	<0.0001
Education (≥high school, %)	11,971 (77.6)	9889 (78.9)	10,490 (71.8)	<0.0001	18,570 (66.3)	17,807 (63.9)	14,689 (55.1)	<0.0001
Monthly income (≥2 million KRW, %)	10,596 (77.3)	8632 (77.7)	8425 (69.6)	<0.0001	17,408 (71.2)	16,362 (69.6)	12,627 (60.2)	<0.0001

HbA1c, hemoglobin A1c; hs-CRP, high sensitive C-reactive protein; KRW, Korean won. Values are expressed as means (SE) or percentage. The *p*-trend was obtained in general linear model analysis and Cochran–Mantel–Haenszel analysis, with adjustment for age.

**Table 2:** Odds ratios (95% confidence intervals) for diabetes by tertiles of stress levels.

Variables	Men				Women			
	T1 (Lowest)	T2 (Intermediate)	T3 (Highest)		T1 (Lowest)	T2 (Intermediate)	T3 (Highest)	
	(*n* = 15,562)	(*n* =12,637)	(*n* = 14,746)	*p*-Trend	(*n* = 28,227)	(*n* = 28,132)	(*n* = 26,975)	*p*-Trend
Prevalence (%)	1660 (10.7)	1374 (10.9)	1883 (12.8)		1472 (5.2)	1673 (6.0)	1929 (7.2)	
Unadjusted OR (95% CI)	1.00	1.02 (0.95–1.10)	1.23 (1.14–1.32)	<0.0001	1.00	1.15 (1.07–1.24)	1.40 (1.31–1.50)	<0.0001
Adjusted OR (95% CI)	1.00	1.07 (0.99–1.17)	1.34 (1.24–1.45)	<0.0001	1.00	1.12 (1.03–1.21)	1.29 (1.19–1.40)	<0.0001

ORs (95% CIs) were conducted by logistic regression model. Adjusted ORs (95% CIs) were obtained with adjustment for age, body mass index, alcohol intake, smoking, exercise, education level, income status, and energy intake. The *p*-trend was obtained in general linear model analysis with adjustment for age, body mass index, alcohol intake, smoking, exercise, education level, income status, and energy intake.

**Table 3:** Dietary intakes of the subjects by tertiles of stress levels.

Variables	Men				Women			
	T1 (Lowest)	T2 (Intermediate)	T3 (Highest)		T1 (Lowest)	T2 (Intermediate)	T3 (Highest)	
	(*n* = 15,562)	(*n* =12,637)	(*n* = 14,746)	*p*-Trend	(*n* = 28,227)	(*n* = 28,132)	(*n* = 26,975)	*p*-Trend
Energy (kcal)	1891.4 ± 4.05	1856.5 ± 4.38	1827.5 ± 4.34	<0.0001	1742.6 ± 2.99	1706.1 ± 2.99	1659.8 ± 3.25	<0.0001
Protein (g)	63.3 ± 0.1	62.5 ± 0.1	61.5 ± 0.1	<0.0001	58.4 ± 0.1	57.4 ± 0.1	56.6 ± 0.1	<0.0001
Fat (g)	30.3 ± 0.1	30.3 ± 0.1	30.1 ± 0.1	0.026	26.6 ± 0.1	26.2 ± 0.1	25.8 ± 0.1	0.001
Carbohydrate (g)	386.5 ± 0.3	387.1 ± 0.3	388.2 ± 0.3	<0.0001	304.1 ± 0.2	305.5 ± 0.2	306.7 ± 0.2	<0.0001
Fiber (g)	5.9 ± 0.02	5.7 ± 0.02	5.5 ± 0.02	<0.0001	6.0 ± 0.01	5.8 ± 0.01	5.7 ± 0.01	<0.0001
Vitamin A (μg RE)	500.5 ± 2.4	476.1 ± 2.4	466.6 ± 2.4	<0.0001	492.6 ± 1.7	475.2 ± 1.7	468.9 ± 1.8	<0.0001
Vitamin C (mg)	102.9 ± 0.4	98.0 ± 0.4	92.0 ± 0.4	<0.0001	116.8 ± 0.3	111.8 ± 0.3	107.7 ± 0.4	<0.0001
Vitamin E (mg)	8.3 ± 0.02	8.1 ± 0.02	8.0 ± 0.02	<0.0001	8.2 ± 0.02	8.0 ± 0.02	8.0 ± 0.02	0.044
Grains (g)	752.2 ± 1.0	760.1 ± 1.1	774.8 ± 1.1	<0.0001	654.2 ± 0.8	664.9 ± 0.8	677.9 ± 0.9	<0.0001
Rice and other cereals (g)	653.7 ± 1.1	658.0 ± 1.3	668.1 ± 1.3	<0.0001	573.2 ± 0.9	581.5 ± 0.9	591.3 ± 1.0	<0.0001
Noodles and breads (g)	96.2 ± 1.3	106.5 ± 1.6	121.0 ± 1.6	<0.0001	77.5 ± 0.9	86.7 ± 1.1	101.9 ± 1.3	<0.0001
Potatoes (g)	19.5 ± 0.3	19.0 ± 0.4	19.3 ± 0.4	0.796	24.4 ± 0.2	24.6 ± 0.2	25.1 ± 0.2	0.001
Vegetables (g)	312.8 ± 1.4	297.9 ± 1.5	291.5 ± 1.5	<0.0001	310.6 ± 1.0	301.7 ± 1.0	298.7 ± 1.1	<0.0001
Fruits (g)	156.2 ± 1.2	146.0 ± 1.3	129.7 ± 1.2	<0.0001	215.7 ± 1.1	203.7 ± 1.1	187.9 ± 1.1	<0.0001
Protein foods (g)	144.8 ± 0.6	142.8 ± 0.6	137.4 ± 0.6	<0.0001	129.4 ± 0.4	124.8 ± 0.4	120.0 ± 0.4	<0.0001
Meats (g)	60.0 ± 0.4	61.0 ± 0.5	59.9 ± 0.5	0.268	46.4 ± 0.3	45.6 ± 0.3	45.8 ± 0.3	0.310
Fish and shellfish (g)	43.2 ± 0.3	41.0 ± 0.3	39.2 ± 0.3	<0.0001	41.5 ± 0.2	40.1 ± 0.2	38.6 ± 0.2	<0.0001
Eggs (g)	15.0 ± 0.3	15.4 ± 0.3	16.1 ± 0.3	0.012	17.2 ± 0.3	17.0 ± 0.2	17.6 ± 0.3	0.001
Beans and products (g)	31.5 ± 0.2	31.5 ± 0.2	30.6 ± 0.2	0.009	32.5 ± 0.1	31.9 ± 0.1	31.5 ± 0.2	0.343
Milk and dairy products (g)	229.5 ± 14.5	232.6 ± 19.0	236.7 ± 14.1	0.769	203.0 ± 3.4	204.3 ± 3.0	230.0 ± 3.7	<0.0001
Dietary variety score	58.4 ± 0.1	58.9 ± 0.1	56.9 ± 0.1	<0.0001	57.0 ± 0.1	57.1 ± 0.1	55.3 ± 0.1	<0.0001

RE, retinol equivalent. Values were expressed as means (SE) and adjusted for total energy intake by using the residual method. The *p*-trend was obtained in general linear model analysis with adjustment for age, body mass index, alcohol intake, smoking, exercise, education level, and income status.

**Table 4:** Odds ratios (95% confidence intervals) for diabetes by tertiles of stress levels and dietary variety score.

Variables	Men				Women			
	T1 (Lowest)	T2 (Intermediate)	T3 (Highest)	*P*_stress_ for Trend	T1 (Lowest)	T2 (Intermediate)	T3 (Highest)	*P*_stress_ for Trend
	(*n* = 15,562)	(*n* =12,637)	(*n* = 14,746)		(*n* = 28,227)	(*n* = 28,132)	(*n* = 26,975)	
**Dietary variety score**								
**T3 (Highest)**								
Prevalence (%)	403 (7.8)	387 (8.6)	550 (11.6)		320 (3.5)	390 (4.1)	398 (4.8)	
Adjusted OR (95% CI)	1.00 (ref)	1.14 (0.98–1.34)	1.64 (1.42–1.90)	<0.0001	1.00 (ref)	1.14 (0.97–1.34)	1.31 (1.11–1.55)	0.002
**T2 (Intermediate)**								
Prevalence (%)	634 (11.3)	482 (11.0)	561 (12.3)		537 (5.3)	564 (5.9)	550 (6.6)	
Adjusted OR (95% CI)	1.34 (1.17–1.55)	1.37 (1.17–1.59)	1.65 (1.42–1.91)	0.002	1.28 (1.10–1.49)	1.37 (1.18–1.60)	1.55 (1.32–1.81)	0.006
**T1 (Lowest)**								
Prevalence (%)	623 (13.1)	505 (13.4)	772 (14.1)		615 (6.9)	719 (8.0)	981 (9.5)	
Adjusted OR (95% CI)	1.47 (1.27–1.71)	1.62 (1.38–1.89)	1.83 (1.58–2.12)	0.001	1.36 (1.17–1.59)	1.59 (1.37–1.86)	1.85 (1.59–2.15)	<0.0001
***P*_DVS_ for trend**	<0.0001	<0.0001	0.094		0.0009	<0.0001	<0.0001	

DVS, dietary variety score. OR, odds ratio; CI, confidence interval. ORs (95% CIs) were conducted by logistic regression model. Adjusted ORs (95% CIs) were obtained with adjustment for age, body mass index, alcohol intake, smoking, exercise, education level, income status, income status, and energy intake. The *p*-trend was obtained in general linear model analysis with adjustment for age, body mass index, alcohol intake, smoking, exercise, education level, income status, and energy intake.
